# Recovery of Agricultural Odors and Odorous Compounds from Polyvinyl Fluoride Film Bags

**DOI:** 10.3390/s100908536

**Published:** 2010-09-13

**Authors:** David B. Parker, Zena L. Perschbacher-Buser, N. Andy Cole, Jacek A. Koziel

**Affiliations:** 1 USDA-ARS, U.S. Meat Animal Research Center, Clay Center, NE, 68933 USA; 2 Adams State College, Alamosa, CO, 81102 USA; E-Mail: zenabuser@adams.edu; 3 USDA-ARS, Conservation and Production Research Laboratory, Bushland, TX, 79012 USA; E-Mail: andy.cole@ars.usda.gov; 4 Iowa State University, Ames, IA, 50011 USA; E-Mail: koziel@iastate.edu

**Keywords:** odor sampling, gas chromatography-mass spectrometry, Tedlar, volatile fatty acid, odor detection threshold, volatile organic compound, single-compound odor threshold, animal feeding operation, odor activity value

## Abstract

Accurate sampling methods are necessary when quantifying odor and volatile organic compound emissions at agricultural facilities. The commonly accepted methodology in the U.S. has been to collect odor samples in polyvinyl fluoride bags (PVF, brand name Tedlar®) and, subsequently, analyze with human panelists using dynamic triangular forced-choice olfactometry. The purpose of this research was to simultaneously quantify and compare recoveries of odor and odorous compounds from both commercial and homemade PVF sampling bags. A standard gas mixture consisting of p-cresol (40 μg m^−3^) and seven volatile fatty acids: acetic (2,311 μg m^−3^), propionic (15,800 μg m^−3^), isobutyric (1,686 μg m^−3^), butyric (1,049 μg m^−3^), isovaleric (1,236 μg m^−3^), valeric (643 μg m^−3^), and hexanoic (2,158 μg m^−3^) was placed in the PVF bags at times of 1 h, 1 d, 2 d, 3 d, and 7 d prior to compound and odor concentration analyses. Compound concentrations were quantified using sorbent tubes and gas chromatography/mass spectrometry. Odor concentration, intensity, and hedonic tone were measured using a panel of trained human subjects. Compound recoveries ranged from 2 to 40% after 1 h and 0 to 14% after 7 d. Between 1 h and 7 d, odor concentrations increased by 45% in commercial bags, and decreased by 39% in homemade bags. Minimal changes were observed in intensity and hedonic tone over the same time period. These results suggest that PVF bags can bias individual compound concentrations and odor as measured by dynamic triangular forced-choice olfactometry.

## 1. Introduction

In the U.S., emissions from animal feeding operations (AFOs) primarily focus on inventories of ammonia, hydrogen sulfide, and particulate matter due to national air quality standards regulations. Air quality complaints near AFOs are generally associated with emissions of odors; however, there are no national regulations on their emissions. In fact, only a small number of states in the U.S. currently have odor regulations in place [1,2]. Currently, odor is assessed using either field or laboratory olfactometry. With field olfactometry, odor is assessed using human panelists and a portable dilution device such as the Nasal Ranger® (St. Croix Sensory Inc., Lake Elmo, MN, USA) or Barnebey-Sutcliffe scentometer (Barnebey-Sutcliffe Corp., Columbus, OH, USA). With laboratory olfactometry, odor samples are collected in plastic field sampling bags and returned to the laboratory for analysis. The standard laboratory method for quantifying odor concentration with human panelists is dynamic triangular forced-choice olfactometry (DTFCO) [3]. The dilution ratio of clean air to odorous air at which the panelist detects but does not recognize the odor is the detection threshold (DT) [4,5].

PVF sampling bags are the standard in U.S. odor laboratories because of their low cost and reported non-reactive, chemically inert qualities [6]. The PVF film used for making commercial sampling bags is manufactured by DuPont™ under the Tedlar® brand name [7,8], and the bags are commonly called ‘Tedlar bags’ by practitioners. As compared to American odor laboratories, most European and Australian odor laboratories currently use polyethyeneterephthalate (brand name Melinex® or Nalophan®) bags [9], while polyester and Nalophan bags are often used in Japan [10,11]. Commercial Tedlar bags can be purchased in the U.S. for $10 to 20 per bag, while homemade bags can be made for about $3.20/bag [12].

Several scientists have expressed concern about the integrity of concentrations, both odor and chemical, at the time of analysis using Tedlar sampling bags [13–15]. Zhang *et al.* [16] found poor correlations between field-measured odor concentrations using Nasal Rangers and odor concentration measured with Tedlar sample bags and DTFCO, and Keener *et al.* [13] reported background odorants in Tedlar sampling bags at concentrations that affected olfactory analysis. However, Parker *et al.* [6] found odor intensities correlated well with the laboratory DTFCO measurements and reported heat-treating the Tedlar bags at 100 °C reduced background DTs to an acceptable level.

Volatile fatty acids (VFAs) and reduced sulfur compounds are important odor components of agricultural, industrial, and municipal wastewater treatment systems. In addition, both phenol and indole compounds are important odorants associated with AFOs. VFA recoveries are affected by storage time [14,15], while those of indole compounds are affected immediately upon sampling [13,14]. Koziel *et al.* [17,18] compared recoveries of standard gases using Tedlar, Teflon, foil, and Melinex sampling bags. Melinex bags had the highest recoveries (71.7% and 47.2%) at 0.5 and 24 h, respectively, followed by Teflon (75.4% and 39.4%), homemade Tedlar (47.3% and 37.4%), commercial Tedlar (67.6% and 22.7%), and foil (16.4% and 4.3%) bags.

Mochalski *et al.* [19] compared Nalophan, transparent Tedlar, black Tedlar, Teflon, and Flexfoil (SKC, Inc., Eighty Four, PA, USA) bags for storage of sulfur compounds such as hydrogen sulfide, methanethiol, and dimethyl sulfide. Mochalski *et al.* [19] concluded that none of the bags were adequate for long-term storage, but that Flexfoil bags were the best for storage up to 24 h.

While considerable research has been conducted to evaluate recovery of odorous compounds from odor sampling bags, little research has reported odor recovery using DTFCO. Additionally, odorous compound recoveries have been compared to individual references for single-compound odor thresholds (SCOT), while the scientific literature suggests a broad range of SCOT. With these previous limitations in mind, the specific objectives of this research were to:

1. Calculate and compare published SCOT values for individual odorous compounds using several measures of central tendency (median, arithmetic mean, and geometric mean); and
2. Quantify and compare the recovery of odor and odorous compounds in PVF sampling bags at sample storage times ranging from 1 h to 7 d.

## 2. Materials and Methods

### 2.1. SCOT and Odor Activity Value for Individual Compounds

To assess the overall importance of odor and compound recoveries, a comprehensive literature review was conducted to determine the SCOT for individual compounds [4,10,20–37]. Other compilations of odor thresholds were also consulted [38–40]. If the literature presented SCOT in units of parts per billion by volume (ppbv), the concentrations were converted to mass per volume (μg m^−3^). A spreadsheet was constructed for each compound, and the median, mean, and geometric mean SCOT were calculated. If a single reference gave a range of odor thresholds for a specific compound, then the minimum and maximum were used in the SCOT calculations. Odor activity values (OAV), defined as the concentration of the compound divided by the SCOT for that compound [14], were calculated for each compound and time period. For each compound, the geometric mean SCOT was used for calculating the OAV. The total OAV (OAV_SUM_) for a mixture of odorous compounds was calculated by summing the individual compound OAVs.

### 2.2. PVF Bags

Two types of 10 L PVF Tedlar bags were considered in this study: Commercial (C) and Homemade (H). The C bags were purchased from SKC Inc. The homemade bags were constructed of TST20SG4 transparent film purchased directly from Dupont™. The film was cut into lengths of 92 cm, folded lengthwise, then heat sealed using a Vertrod® Model 14OB open-back heat sealer with 0.63 cm seal width and 51 cm maximum seal length (Therm-O-Seal, Mansfield, TX, USA). As part of the H bag-making process, bags were filled with ultra-pure odor-free air and heat treated in a laboratory drying oven at 100 °C for 24 h to remove residual odors from Tedlar off-gassing [6].

### 2.3. Standard Gas Generators

Standard gases were generated with a continuous generator using permeation technology as described by others [41]. The seven VFAs were generated with a model 491 M standard continuous gas generator (Kintek, LaMarque, TX, USA) and a permeation oven temperature of 80 °C, while the aromatic compound p-cresol (4-methylphenol) was generated in a homemade continuous gas generator using a permeation oven temperature of 50 °C. The homemade generator consisted of a 1 L glass container, a heating mantle (Glas-Col, Terre Haute, IN, USA), a ±0.2 °C temperature controller (Cole Parmer, Vineland, NJ, USA), and a 0–5 L/min mass flow controller (Aalborg, Orangeburg, NY, USA). The two gas streams were combined, and the resultant gas stream quantified with sorbent tube sampling and subsequent gas chromatography/mass spectrometry (GC/MS) analysis. The combined standard gas used in this research had the following concentrations: VFAs, acetic (2,311 μg m^−3^), propionic (15,800 μg m^−3^), isobutyric (1,686 μg m^−3^), butyric (1,049 μg m^−3^), isovaleric (1,236 μg m^−3^), valeric (643 μg m^−3^), hexanoic (2,158 μg m^−3^); and aromatics, p-cresol (40 μg m^−3^).

### 2.4. Experimental Design

The experiment was designed such that all samples in the entire experiment could be analyzed by a single odor panel at the West Texas A&M University (WTAMU) olfactometry laboratory. The experiment was designed to reduce and eliminate variability between odor panels, which has been documented to be as high as 22–50% [5,42]. Thus, the experiment was designed under the limitation of the number of samples that could be conducted in a single odor panel by the olfactometry laboratory in a 4-h period, usually 8–10 odor samples. Odor laboratories try not to analyze more than 8–10 samples in a single period to avoid panel fatigue and potential bias in analytical results.

A Tedlar bag of each type, C and H, was filled with the standard gas mixture from the continuous gas generator at times of 7 d, 3 d, 2 d, 1 d, and 1 h prior to volatile organic compound (VOC) and olfactometry testing, for a subtotal of 10 Tedlar bags (2/treatment) filled with the standard gas. Bags were filled with the standard gas mixtures, purged once, then filled again. An additional bag of each type was also filled with odor-free air 1 h prior to olfactometry testing. These two ‘blank’ bags were filled with the odor-free air, purged once, and then filled again to follow the same filling procedure used for the standard gas bags.

Just prior to olfactometry testing, a subsample of the air in the bags was sampled for chemical analysis. Air (600 mL, 100 mL min^−1^ for 6 min) was pulled by vacuum from the bags into stainless steel sorbent tubes (90 mm × 5 mm I.D., SKC, Inc.) filled with 150 mg Tenax TA sorbent using an SKC Pocket Pump® (SKC, Inc.). A total of 12 Tedlar bags were analyzed in the experiment.

### 2.5. Laboratory Dynamic Dilution Olfactometry

Olfactometry analysis was performed per ASTM Standards [3,43,44] on d 7 of the experiment, analyzing odor in sample bags that had been maintained at room temperature for 1 h, 1 d, 2 d, 3 d, and 7 d. All human panelists were previously trained in all aspects of the odor analysis. Panelists were selected based on sensitivity to the n-butanol reference gas as described in the ASTM standards, with those individuals considered hypernosmic or anosmic excluded. The panel consisted of four human panelists, who sniffed each sample twice, yielding a total of eight individual olfactometry readings per sample. A machine blank was also analyzed prior to analysis to verify the absence of detectable background odor originating from the olfactometer.

Samples were analyzed for odor concentration (detection threshold, DT, also called “dilutions to threshold”) using a triangular forced-choice olfactometer (AC’SCENT International Olfactometer, St. Croix Sensory, Inc.). Individual panelist DTs were calculated according to ASTM standards [3] as the geometric mean of the concentration at which the last incorrect guess occurred and the next higher concentration where the odor was correctly detected. Samples were analyzed for intensity using a static-scale method by comparison to five standard n-butanol solutions, following the general guidelines of ASTM [43]. Solutions were placed into five capped flasks at concentrations of 0.25, 0.75, 2.25, 6.75, and 20.25 ml n-butanol per L of water, which corresponded to intensities of 1.0, 2.0, 3.0, 4.0, and 5.0, respectively [5]. Panelists would alternate between sniffing the headspace of the standard n-butanol/water solutions and the odor sample, then assign an intensity that most closely matched one of the five standard solutions. The average intensity was calculated for the panel using the arithmetic mean. Panelists would assign a hedonic tone (HT) rating in a similar manner by sniffing the full strength odor sample. Panelists were asked to subjectively assign a score for HT on a scale of −4 to +4, with −4 being very unpleasant, 0 being neutral, and +4 being very pleasant [5]. The average HT was calculated for the panel using the arithmetic mean. Percent recoveries of chemical compounds were calculated as the amount recovered at time (T) divided by the initial concentration of the standard gas.

### 2.6. GC/MS with Thermal Desorption

Sorbent tube samples were analyzed using an automated thermal desorber (ATD) (Perkin-Elmer, Shelton, CT, USA) and a Varian 3,800/Saturn 2,000 gas chromatograph (GC) equipped with a mass spectrometer (MS) (Varian, Inc., Walnut Creek, CA, USA). Samples were automatically desorbed in the ATD at 225 °C for 15 min, trapped in a quartz cryotrap at −30 °C, then heated to 225 °C and injected into the GC/MS. Upon injection, samples were held at 225 °C for 20 min with 1 mL/min flow of helium. The GC column was ramped from 60 to 230 °C at a rate of 6 °C/min for a total run time of 30.3 min.

Chemicals for the VFA standards were mixed in hexanes, and the p-cresol standard was mixed in methanol. All chemicals and solvents were FCC kosher grade (Sigma Aldrich, St. Louis, MO, USA). Standards were prepared using serial dilutions, then injected onto clean tubes using a calibration solution loading rig (CSLR, Markes International Inc., Wilmington, DE, USA). The liquid calibration standard was introduced through the CSLR injector septum in argon carrier gas using a standard GC syringe. A minimum of six concentrations with one replicate at each concentration were used to construct the standard curves. In addition, seven replicates were conducted at two of the lower concentrations for calculation of method detection limit (MDL). MDLs were calculated per U.S. EPA guidelines as the product of the standard deviation of seven replicates and the Student’s t-value at the 99% confidence level (Table 1). Standard curves were fit using linear regression with the curve forced through the origin. All sorbent tubes were conditioned with the ATD and analyzed on the GC/MS prior to sampling to verify that clean tubes were used in the experiment.

### 2.7. Statistical Analyses

Standard regression and correlation techniques were used to model changes in concentrations with T and to test for statistical significance (α = 0.05). Graphing was performed using Microsoft Excel, and statistical analyses were performed using SPSS Version 17 [45].

## 3. Results and Discussion

### 3.1. SCOT Values

SCOT values are presented in Table 2 for the seven VFAs, p-cresol, and three other aromatic compounds commonly found near agricultural facilities. However, because of the variation in magnitude of published SCOT, it becomes difficult to quantitatively assess the overall importance of the compound concentrations in the bags. There was considerable difference between SCOT calculated using the arithmetic mean, geometric mean, or median. Median and geometric mean SCOT were lowest and similar, whereas the arithmetic mean SCOT was typically greater by an order of magnitude or more (Table 2). This was a result of the arithmetic mean being influenced by the larger individual values. Due to odor thresholds presented in the literature not always being differentiated as to whether they are detection or recognition thresholds, it is probable that some of the higher SCOT values are actually recognition thresholds.

### 3.2. Background Bag Odor and Compound Concentrations

Several chemical compounds were detected in the blank bags analyzed 1 h after filling with odor-free air. The most predominant peaks on the chromatograph were N, N-dimethylacetamide (DMAC) and phenol (concentrations not quantified). Acetic acid was quantified at 203 and 73 μg m^−3^ in H and C bags, respectively. Tedlar off-gassing was the source of compounds detected, as compound concentrations in the odor-free air used to fill the bags were well below detection limits for all compounds (data not shown). The DT as determined by laboratory olfactometry for these same blank bags was 8 and 19 for H and C bags, respectively. The OAV for acetic acid was 0.44 and 0.16 for H and C bags, respectively. These results are similar to others reporting measurable background odor and compound concentrations in blank Tedlar bags [6,13,14,17,46,47]. DTs of 20–60 have been reported in blank Tedlar bags 24 h after filling with clean air, with DTs as high as 200 from blank Tedlar bags that were heated in the oven then analyzed without purging [6].

### 3.3. Chemical Compound Recoveries

Percent recoveries of individual compounds varied with T and the type of Tedlar bag (Table 3). Percent recoveries for all eight VOCs averaged about 16 to 18% at 1 h and 2–5% at 7 d. At 1-h post sampling, the highest recoveries were found for isobutyric and isovaleric acids. For these four-and five-carbon compounds, their branched-chain isomers were recovered at higher percentages than the straight-chain isomers. For example, the average recovery of both bag types was 39.2% for isobutyric acid and 18.2% for butyric acid, or 53.5% lower for the straight-chain isomer. Likewise, recovery of isovaleric acid averaged 27.3% and valeric acid averaged 4.8%, or 82.4% lower for the straight-chain isomer. The straight chain isomers with low volatility and vapor pressure [48] have lower recovery rate than the branched isomers due to enhanced adsorptivity.

The aromatic compound p-cresol had the lowest recovery at 1 h. It was also the least volatile and most adsorptive compound of those analyzed. Ironically, p-cresol has been implicated as one of the most important odorous compounds at distances greater than several kilometers downwind of AFOs [49–51]. Thus, the use of Tedlar bags would be inappropriate for quantification of p-cresol or other aromatic compounds under ambient conditions downwind of AFOs.

For all VOCs, it was evident that most of the VOCs were lost within the first hour after sampling, with VOC concentrations continuing to decrease until completion of the experiment. These results are similar to those of others who reported a 50% decrease in concentrations of VOCs associated with tobacco odors between 4 and 30 h after sampling in Nalophan® bags [7]. Acetic acid and p-cresol were the only compounds that did not continue to decrease until day 7, but this was for the C bags only. As shown in the table of regression coefficients for individual VOCs, these two anomalies were the only regressions with positive slopes and highly non-significant regressions (*p* ≥ 0.56) (Table 4). Ten of the remaining 14 regressions were significant.

**Figure 1.**
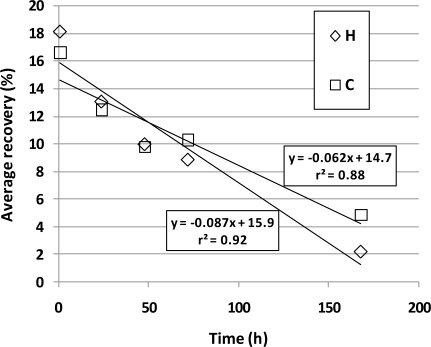
Average recovery of eight VOCs over a period of 168-h (7-d) since time of filling for homemade (H) and commercial (C) Tedlar PVF bags.

Regression analyses of the average percent recoveries of Table 3 are presented in Figure 1. A strong downward linear trend was observed for both H (r^2^ = 0.92, *p* = 0.009) and C (r^2^ = 0.88, *p* = 0.018) bags. As compared to the C bags, the H bags had slightly higher average percent recoveries at 1 h, 1 d, and 2 d, indicating that H bags may be more appropriate for VOC analyses if samples were analyzed quickly. But the steeper slope indicates a faster breakdown or loss of VOCs in H bags. Thus, C bags would be more appropriate if the sample could not be analyzed until 3 d or longer after sampling. However, both bag types demonstrated poor recoveries of VOCs, even if analyzed as early as 1 h post sampling. These results suggest that Tedlar bags make a poor storage device when sampling for individual VFA or p-cresol, and for accurate quantification of these compounds, an alternative method such as sorbent tubes should be used.

### 3.4. OAV

Like individual VOC concentrations, calculated OAVs also decreased with time. The initial OAV_SUM_ value (using geometric mean SCOT) for the standard gas was 620, and decreased to 139 and 136 at 1 h for H and C bags, respectively (Table 5). Even at 1 h, the OAVs were only 22.4 and 21.9% of the original OAV for H and C bags, respectively. At day 7, six of the eight compounds had OAVs less than 1.0, and the total OAV (OAV_SUM_) was only 7.0 and 18.3 for H and C bags, respectively. This equates to 1.1 and 2.9% of the original OAV_SUM_, and compares similarly to average percent recovery of all eight VOCs of 2.2 and 4.9% (Table 3).

The OAV values for p-cresol were less than 1.0 at all sampling T. This indicates that although it had an OAV greater than 1.0 when placed in the bag, p-cresol did not likely contribute to odor perception after being placed in the Tedlar bag unless it reacted to form other odorous compounds.

The calculated OAVs reported herein are considerably higher than those reported by others [14], and the difference should be explained. Trabue *et al.* [14] reported a total OAV (OAV_SUM_) of 10.6 for a standard gas mixture and 42.7 for a swine production facility, as compared to an OAV_SUM_ value of 620 for the standard gas mixture. The reason for the major difference is in the method of calculation, and more specifically, the SCOT used for the individual compounds. Trabue *et al.* [14] used individual SCOT published by Devos *et al.* [39], which were considerably higher than the calculated geometric mean SCOT presented in Table 2 and used for subsequent calculation of OAV of this research. For this reason, readers are cautioned about comparing the absolute magnitude of OAV or OAV_SUM_ from this research to any other research projects without first evaluating and comparing the individual compound SCOT used to calculate the OAV.

**Figure 2.**
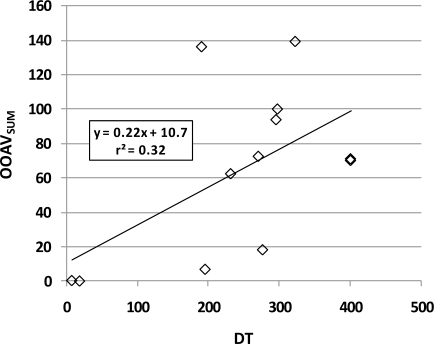
An example of the poor correlation between the sum of calculated odor activity values for eight compounds (OAV_SUM_, from Table 5) and odor concentration (DT, from Table 6).

Individual OAV is highly dependent on SCOT, which also highly variable in the literature. Calculated OAV_SUM_ depends not only on SCOT but also on the statistical method used for calculating the central tendency. A comparison of OAV_SUM_ calculated using geometric mean SCOT values (Table 5) to measured DTs for all odor samples from this research shows a slight but non-significant positive correlation (Figure 2, r^2^ = 0.32; *p* = 0.056). If the median SCOT were used instead of the geometric mean SCOT, OAV_SUM_ for the standard gas would be 782 and a similar but different graph would be produced (y = 0.23x + 12.4, r^2^ = 0.30, *p* = 0.067; graph not shown). Likewise, if the arithmetic mean SCOT were used, the OAV_SUM_ of the standard gas would be much less, only 13.4, and the resulting linear model would be: (y = 0.0063x + 0.58, r^2^ = 0.28, *p* = 0.077; graph not shown).

The OAV calculation presented above assumes a simple additive concept when combining individual odorants. It is important to note that other researchers have reported additive, antagonistic, and synergistic relationships [52–55].

### 3.5. Laboratory Dynamic Dilution Olfactometry Odor Recoveries

**Figure 3.**
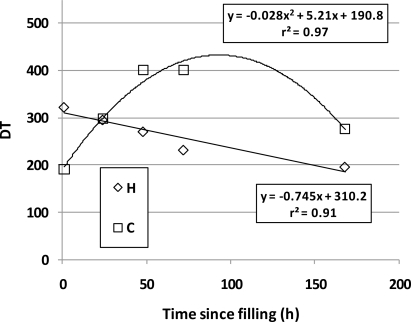
An example of how odor concentration (DT) as measured by laboratory dynamic triangular forced-choice olfactometry (DTFCO) changes with time in homemade (H) and commercial (C) Tedlar PVF bags, starting at time 1-h post filling. Homemade bags exhibited a linear relationship with time, while commercial bags exhibited a quadratic relationship.

Odor concentrations as measured using DTFCO are presented in Table 6. As opposed to individual compound concentrations, for DTFCO the original odor concentration (DT) is never known because the sample must be placed in a Tedlar, or other type of bag, for analysis. Thus, percent recoveries cannot be calculated similar to what is presented in Table 3. A comparison of odor concentration *vs.* T for the H and C bags revealed a strong negative linear trend between DT and T for the H bags (r^2^ = 0.91, *p* = 0.012), whereas a quadratic trend was observed for the C bags (Linear: r^2^ = 0.03, *p* = 0.79; Quadratic: r^2^ = 0.97, *p* = 0.032) (Figure 3). It is likely that the continued off-gassing from C bags contributed to the increase in DT over the 2 to 3 day period after sampling. Trabue *et al.* [14] reported a similar increase in several VOCs between 0.5- and 72-h post-sampling. The H bags were heat-treated to minimize odorous effects of the solvent used in the Tedlar manufacturing process, which most likely explains the difference in the shapes of the two curves in Figure 3.

Minimal changes were observed in intensity and hedonic tone over the same time period (Table 6). Intensities ranged from 3.1 to 3.5 for the H bags, and 2.1 to 3.1 for C bags, with no distinguishable trends over time. Findings were similar for hedonic tone. The observed changes in odor concentration (DT), intensity, and hedonic tone changed little compared to the actual VOC concentrations, which decreased considerably over the same time period. According to the extremely low VOC recoveries and corresponding low OAV_SUM_ for the 7-d samples (Tables 3 and 5), one would expect a similarly low odor concentration (DT). However, the DTs for both H and C bags at 7 d were relatively similar to those measured at 1-h post sampling.

The combination of findings, including (1) the quadratic relationship between DT and T in Figure 3; (2) the lack of correlation between OAV_SUM_ and DT in Figure 2; (3) the discrepancy between the small 7-d percent recoveries of Table 3; and (4) the minimal differences in DT with time of Table 6 altogether lead to a possible yet unproven hypothesis. It is possible that either the VOCs in the sample air reacted with the solvent off-gassed from the Tedlar bag to form other odorous VOCs, or that the combination of odorants in the gaseous sample and the off-gassed VOCs produced a synergistic effect to the odor panelists. If this were not occurring, then the DT as measured by DTFCO at 7 d should have been similar to that for the bag filled with odor-free air (DT = 8 and 19), as compared to that measured on the standard gas VOC sample at 7 d (DT = 196 and 277). Thus, one explanation is that odorous compounds were present in the 7 d Tedlar bags other than those eight VOCs quantified in this research. Thus, while the overall DT as measured by DTFCO changed only marginally with time, the chemical compounds that contributed to the change may not have been the same chemical compounds that were introduced into the bag at time zero. As scientists and engineers who rely on accurate sampling methods for making conclusions about abatement measures and regulatory decisions, this bias should be accounted for in any important decision process. An example is the recommendation made by Qu and Feddes [56], who suggested that Tedlar bags should only be used in DTFCO when odor concentrations are greater than 608 odor units (DT), such as those found within a swine barn.

## 4. Conclusions

The following conclusions were drawn from this research:

1. A compilation of published SCOT values shows that they can span one to two orders of magnitude. This makes calculation of OAVs highly dependent on SCOT. Median and geometric mean SCOT were of lower and similar magnitude than the higher arithmetic mean SCOT.
2. Low chemical compound recoveries were observed in both H and C Tedlar sampling bags, averaging about 16 to 18% at 1 h and from 2 to 5% at 7 d post sampling. Likewise, odor measured by human panelists using DTFCO changed with time, most likely because of chemical adsorption in the Tedlar bags and/or chemical reactions among the different odorous compounds. Because of this sampling bias, researchers and practitioners are cautioned to account for the extreme losses in odorous compounds when using Tedlar bags for quantification of VOC or odor emissions.

## Figures and Tables

**Table 1.** Minimum mass, method detection limits, and coefficient of determination statistics for the standard curves.

	**Minimum Mass Used in Standard Curve (ng)**	**MDL (ng)**	MDL* (μg m^−3^)	**Regression (r^2^)**
Acetic acid	1.9	5.6	9.3	0.97
Propionic acid	1.0	4.6	7.7	0.85
Isobutyric acid	1.5	11.3	18.8	0.84
Butyric acid	2.5	5.4	9.0	0.90
Isovaleric acid	0.06	0.17	0.28	0.99
Valeric acid	0.6	1.9	3.2	0.97
Hexanoic acid	25	188	313	0.98
*p*-Cresol	0.9	0.88	1.5	0.97

* MDL calculated with sampling volume of 0.6 L.

**Table 2.** A statistical summary of published single-compound odor thresholds (SCOT) for seven volatile fatty acids and four aromatic compounds (μg m^−3^).

**Compound**	N*	**Minimum**	**Maximum**	**Arithmetic Mean**	**Standard Deviation**	**Geometric Mean**	**Median**
Acetic Acid	27	15	352,822	15,970	27,026	467	363
Propionic Acid	25	2.9	30,870	2,170	2,209	101	103
Isobutyric Acid	17	0.8	29,370	1,852	1,032	41	72
Butyric Acid	23	0.4	49,400	7,092	4,605	23	4.4
Isovaleric Acid	17	0.21	1,745	152	99	4.7	6.4
Valeric Acid	18	0.17	34,100	2,640	1,975	11.7	11.5
Hexanoic Acid	16	2.4	17,500	2,265	1,115	83.1	23.7
Phenol	13	10.2	7,700	1,023	583	127	94
p-Cresol	26	0.05	41	10.9	3.0	2.6	4.5
Indole	14	0.02	650	51.8	37.2	1.9	1.9
Skatole	11	0.001	120	30.3	9.2	1.6	0.9

* Some references gave multiple SCOT values, and N denotes number of individual SCOT values used in the statistical analyses. If an individual reference gave a range of SCOT values, then the minimum and maximum values were used in the statistical calculations for overall median, arithmetic mean, and geometric mean odor threshold for each compound.

**Table 3.** Percent recoveries of VOCs in homemade (H) and commercial (C) Tedlar PVF bags filled with the VOC standard gas mixture.

**Odorant**	**Time since filling**									
	**1 h**		**1 d**		**2 d**		**3 d**		**7 d**	

	H	C	H	C	H	C	H	C	H	C
Acetic Acid	19.0	14.2	13.5	9.4	10.6	8.5	8.1	12.7	6.4	14.1
Propionic Acid	21.5	19.4	12.6	13.7	10.0	9.2	8.1	10.3	1.4	4.3
Isobutyric Acid	38.4	40.1	34.3	35.6	29.3	33.0	25.3	31.1	5.4	11.3
Butyric Acid	17.9	18.6	11.7	12.4	8.0	8.6	6.2	8.0	1.0	2.0
Isovaleric Acid	27.0	27.7	18.0	20.2	13.9	13.5	12.0	12.7	0.3	1.5
Valeric Acid	4.7	4.9	3.3	2.3	1.9	1.8	1.5	1.6	0.3	0.2
Hexanoic Acid	14.5	3.7	9.7	2.4	4.9	1.4	8.2	2.1	2.6	0.5
p-Cresol	2.4	4.5	1.6	3.9	1.3	2.2	1.5	3.9	0.0	4.9
	
Mean	18.2	16.6	13.1	12.5	10.0	9.8	8.9	10.3	2.2	4.9

**Table 4.** Regression coefficients for the individual VOC recoveries of Table 3, using the linear model Y = B_0_ + B_1_X where Y = percent recovery and X = time (h).

**Odorant**	**Homemade**					**Commercial**				
	**B_0_**	**B_1_**	**r^2^**	***p***		**B_0_**	**B_1_**	**r^2^**	***p***	
Acetic Acid	15.6	−0.066	0.73	0.065		10.9	0.015	0.12	0.559	
Propionic Acid	17.2	−0.104	0.84	0.028	*	16.2	−0.078	0.81	0.036	*
Isobutyric Acid	38.9	−0.198	0.99	<0.001	***	40.8	−0.170	0.98	0.001	***
Butyric Acid	14.6	−0.090	0.85	0.025	*	15.4	−0.088	0.86	0.024	*
Isovaleric Acid	23.3	−0.145	0.93	0.008	**	24.1	−0.144	0.92	0.010	**
Valeric Acid	3.8	−0.024	0.82	0.032	*	3.6	−0.023	0.74	0.063	
Hexanoic Acid	11.7	−0.059	0.70	0.079		3.0	−0.016	0.76	0.053	
p-Cresol	2.2	−0.013	0.91	0.011	*	3.5	0.006	0.12	0.563	

* Significant at α = 0.05;

** Significant at α = 0.01;

*** Significant at α = 0.001.

**Table 5.** Odor activity values (OAVs) in Homemade (H) and Commercial (C) Tedlar PVF bags filled with the VOC standard gas mixture.

	**Time since filling**										
**Compound**	**0 h**	**1 h**		**1 d**		**2 d**		**3 d**		**7 d**	
	
		H	C	H	C	H	C	H	C	H	C
Acetic Acid	5.0	0.96	0.7	0.7	0.5	0.5	0.4	0.4	0.6	0.3	0.7
Propionic Acid	157.9	34.0	30.6	19.9	21.6	15.8	14.5	12.8	16.3	2.2	6.8
Isobutyric Acid	42.5	16.3	17.1	14.6	15.1	12.5	14.0	10.8	13.2	2.3	4.8
Butyric Acid	46.2	8.3	8.6	5.4	5.7	3.7	4.0	2.9	3.7	0.5	0.9
Isovaleric Acid	269.1	72.6	74.5	48.4	54.4	37.4	36.3	32.3	34.2	0.8	4.0
Valeric Acid	57.0	2.8	2.8	1.9	1.3	1.1	1.0	0.9	0.9	0.2	0.1
Hexanoic Acid	26.7	3.9	1.0	2.6	0.6	1.3	0.4	2.2	0.6	0.7	0.1
p-Cresol	15.8	0.4	0.7	0.2	0.6	0.2	0.3	0.2	0.6	0.0	0.8

OAV_SUM_	620.2	139.3	136.0	93.7	99.8	72.5	70.9	62.5	70.1	7.0	18.2

**Table 6.** Laboratory detection thresholds (DT) as measured by trained human panelists by dynamic triangular forced-choice olfactometry using homemade (H) and commercial (C) Tedlar PVF bags filled with the VOC standard gas (SG) mixture.

Sample storage time	**Odor Concentration (DT, odor units)**		Intensity^*^		Hedonic Tone^**^	

	H	C	H	C	H	C
0 h (initial)	-	-	-	-	-	-
1 h (odor-free air)	8	19	0.8	1.8	0.0	−0.8
1 h (SG)	323	191	3.1	2.8	−2.5	−2.0
1 d (SG)	296	296	3.1	3.1	−2.3	−2.0
2 d (SG)	271	401	3.5	2.1	−2.5	−2.0
3 d (SG)	232	401	3.3	2.5	−2.5	−2.0
7 d (SG)	196	277	3.3	2.3	−2.5	−1.8

* Intensity: Arithmetic means, measured on scale of 1.0 to 5.0 in 1.0 increments, corresponding to headspace concentrations in solutions of 0.25, 0.75, 2.25, 6.75, 20.25 mL n-butanol/L water.

** Hedonic Tone: Arithmetic means, measured on scale of −4.0 to +4.0 in 0.5 increments.
